# Simvastatin Prevents Dopaminergic Neurodegeneration in Experimental Parkinsonian Models: The Association with Anti-Inflammatory Responses

**DOI:** 10.1371/journal.pone.0020945

**Published:** 2011-06-22

**Authors:** Junqiang Yan, Yunqi Xu, Cansheng Zhu, Limin Zhang, Aimin Wu, Yu Yang, Zhaojun Xiong, Chao Deng, Xu-Feng Huang, Midori A. Yenari, Yuan-Guo Yang, Weihai Ying, Qing Wang

**Affiliations:** 1 Department of Neurology, The Third Affiliated Hospital of Sun Yat-Sen University, Guangzhou, Guangdong, People's Republic of China; 2 Centre for Translational Neuroscience, School of Health Sciences, University of Wollongong, New South Wales, Australia; 3 Department of Cardiology, The Third Affiliated Hospital, Sun Yat-Sen University, Guangzhou, People's Republic of China; 4 Department of Neurology, University of California San Francisco and the San Francisco Veterans Affairs Medical Center, San Francisco, California, United States of America; 5 Med-X Research Institute, Shanghai Jiao Tong University, Shanghai, People's Republic of China; Universidad Federal de Santa Catarina, Brazil

## Abstract

**Background:**

In addition to their original applications to lowering cholesterol, statins display multiple neuroprotective effects. N-methyl-D-aspartate (NMDA) receptors interact closely with the dopaminergic system and are strongly implicated in therapeutic paradigms of Parkinson's disease (PD). This study aims to investigate how simvastatin impacts on experimental parkinsonian models via regulating NMDA receptors.

**Methodology/Principal Findings:**

Regional changes in NMDA receptors in the rat brain and anxiolytic-like activity were examined after unilateral medial forebrain bundle lesion by 6-hydroxydopamine via a 3-week administration of simvastatin. NMDA receptor alterations in the post-mortem rat brain were detected by [^3^H]MK-801(Dizocilpine) binding autoradiography. 6-hydroxydopamine treated PC12 was applied to investigate the neuroprotection of simvastatin, the association with NMDA receptors, and the anti-inflammation. 6-hydroxydopamine induced anxiety and the downregulation of NMDA receptors in the hippocampus, CA1(Cornu Ammonis 1 Area), amygdala and caudate putamen was observed in 6-OHDA(6-hydroxydopamine) lesioned rats whereas simvastatin significantly ameliorated the anxiety-like activity and restored the expression of NMDA receptors in examined brain regions. Significant positive correlations were identified between anxiolytic-like activity and the restoration of expression of NMDA receptors in the hippocampus, amygdala and CA1 following simvastatin administration. Simvastatin exerted neuroprotection in 6-hydroxydopamine-lesioned rat brain and 6-hydroxydopamine treated PC12, partially by regulating NMDA receptors, MMP9 (matrix metalloproteinase-9), and TNF-a (tumour necrosis factor-alpha).

**Conclusions/Significance:**

Our results provide strong evidence that NMDA receptor modulation after simvastatin treatment could partially explain its anxiolytic-like activity and anti-inflammatory mechanisms in experimental parkinsonian models. These findings contribute to a better understanding of the critical roles of simvastatin in treating PD via NMDA receptors.

## Introduction

As hydroxymethylglutaryl-coenzyme reductase inhibitors, statins have been widely used to reduce serum low-density lipoprotein (LDL) cholesterol. It has been well established that statins reduce the risk of ischaemic heart disease events and cerebrovascular stroke, and have potential applications in multiple sclerosis, traumatic brain injury, and Alzheimer's disease (AD). Recently, increasing animal and clinical evidence has shown that statins have obvious effects on cognition, dementia and progressive Parkinson's disease (PD), even though conflicting results were observed and the exact mechanisms remain unclear [1]. Anti-inflammatory interventions induced by statins were also observed in various neurological disease models [2]. The application of statins' may have potentially beneficial effects on neuropsychological disorders such as PD.

*N*-methyl-D-aspartate (NMDA) receptors, one of the families of ionotropic glutamate receptors, are widely studied and abundant in the cerebral cortex, hippocampus, nucleus accumbens and striatum [3], [4], [5]. Changes of NMDA receptor populations in the brain are closely associated with many important brain functions, including neuronal apoptosis [6], attention and movement [7] as well as anxiety and depression [8]. Recent studies have demonstrated that NMDA receptors in different brain regions such as the amygdala and hippocampus mediate anxiety and fear-related activity [9], [10]. Mishizen reported that markedly reduced NMDA receptor binding levels were observed in the hippocampus and striatum of aged mice and AD patients [11] in association with the cognitive decline and anxiety. One clinical study by Tsang demonstrated that the NMDA receptor NR2A(N-methyl,D-aspartate receptor subunit 2A subunit)was significantly reduced in the orbitofrontal gyrus of high-anxiety Alzheimer's patients in comparison to low anxiety patients, indicating that changes in the expression of NMDA receptors in the brain may modulate an anxiety-like activity [12]. In addition, overactivation of NMDA receptors is associated with neuronal excitotoxicity leading to cell death [13]. These findings strongly suggest the alterations of brain NMDA receptors may play important roles in neuropsychiatric and movement related disorders.

PD is the second most common neurodegenerative disorder following AD and is characterized by disturbance of the central dopaminergic system and imbalances in some non-dopaminergic systems, including the glutamatergic system. It has been well documented that there is a close interaction between brain glutamatergic NMDA receptors and monoamine dopaminergic systems [14]. Dopaminergic disturbances in the brain may lead to glutamatergic NMDA receptor changes [15] and vice versa [16]. Fiorentini indicated that in the 6-hydroxydopamine-lesioned rat model of PD, D1/NMDA receptor expression was profoundly decreased in the lesioned striatum [17]. Several lines of studies showed that in rodent and primate models of PD NMDA receptor antagonists increased dopaminergic neuronal survival and normalized the levodopa-induced abnormal motor response [18], [19]. Our previous studies and one by Selley [20], [21] have reported that simvastatin profoundly affects D1/D2 dopamine receptors and altered dopamine content in various brain regions, and our recent work has also indicated that simvastatin up-regulates the NMDA receptors in different regions of the rat brain [22]. Increasing evidence shows that inflammatory responses, which are characterized by activation of microglia [23], [24] and accumulation of inflammatory mediators such as inflammatory cytokines and proteases in the substantia nigra and striatum [25], [26], are thought to be responsible for the progression of PD. Hernandez-Romero demonstrated that in LPS-induced PD rats, simvastatin delayed LPS-mediated dopaminergic degeneration via activating the neurotrophic factor BDNF and inhibiting the induction of interleukin-1beta, tumour necrosis factor-alpha, iNOS, mitogen-activated protein kinases, cAMP response element-binding protein, and Akt [27]. Ghosh also found that statins attenuated the activation of both p21(ras) and NF-kappaB in MPP(+)-mediated microglial cells and MPTP-intoxicated mice, accompanying slowing down the progression of dopaminergic neuronal loss and improving motor function [28]. In this study, we sought to determine whether the application of simvastatin influences the expression of NMDA receptors in the PD models and to identify any effects associated with anti-inflammation and anti-excitotoxicity.

To address this issue, we used [^3^H] MK-801 binding autoradiography to determine the response of NMDA receptors to chronic simvastatin treatment across a wide range of brain structures in Parkinsonian rats. Behavioural study was also used to explore the association between the alterations of NMDA receptors and anxiety. In addition, in vitro study was used to investigate the neuroprotection of simvastatin in PC12 cells (Pheochromocytoma 12 Cells)following 6-hydroxydopamine (6-OHDA) neurotoxicity and its association with NMDA receptor and anti-inflammatory responses. This work finds a possible correlation between simvastatin and NMDA receptors based on in vivo and in vitro parkinsonian models.

## Materials and Methods

### Ethics Statement

The animal study has been approved by the University of Wollongong Animal Ethics Committee (project number: AE 08/03) and all animal experiments were conducted in compliance with the *National Institute of Health Guide for the Care and Use of Laboratory Animals (NIH Publications No. 80-23)* revised 1996 guidelines and National Health and Medical Research Council (NHMRC) *Australian Code of Practice for the Care and Use of Animals for Scientific Purposes (2004)*.

### 6-OHDA-Lesioned Parkinsonian Rats and Drug Treatments

Twenty-two male Sprague-Dawley rats (230–250 g) were obtained from the Animal Resources Centre (Perth, Western Australia, Australia) and housed individually in environmentally controlled conditions with *ad libitum* access to standard laboratory chow and water. They were randomized with sixteen rats to create a 6-OHDA-induced parkinsonian treated group, among which eight rats were orally treated with simvastatin (10 mg/kg/day) [21], [22] and eight rats received saline orally. The 6-OHDA lesioned Parkinsonian rat model was performed as described in our previous works [29]. Briefly, male Sprague–Dawley rats (weight 230–250 g) were anesthetized with 75 mg/kg ketamine and 10 mg/kg xylazine (Troy Laboratories Pty, Ltd., Australia). Lesions were performed by unilaterally injecting 6-OHDA into the medial forebrain bundle. The control group received vehicle. One 6-OHDA lesioned rat that received simvastatin orally died after the surgery. After three weeks of 6-OHDA-induced Parkinsonian treatment, rats from each group were sacrificed to examine the NMDA receptor binding.

### Elevated Plus Maze (EPM)

Three weeks after 6-OHDA lesion, rats were tested in the EPM, where the level of anxiety was assessed. The procedure for this test was as described in previous studies [22], [30]. The EPM consists of two open arms (50×7×1 cm) and two closed arms (50×7×30 cm) with an open roof, arranged around a central platform (7×7 cm) so that the arms oppose each other. Light intensity was set at approximately 100 lux along the open arms. A single rat was placed on the central platform facing an open arm and observed for 5 minutes. The number of open and closed arm entries, duration in the open and closed arms and center were scored using a computer program. From these measures, the percentage of time spent in the open arms (100×time open/time open+time closed) and the percentage of open-arm entries (100× time open-arm entries/total entries) were calculated for each animal as the anxiety indexes. Increased time, and/or entries traveled in the open arms of the EPM are interpreted as reduced anxiety-like behavior. The criterion for recording an entry was that the animal had at least half of its body length entered into the arm/center. A rat was considered to be in the central platform zone if its body was positioned in a closed arm and the head and front paw/s were on the central platform.

### Tyrosine Hydroxylase Immunohistochemistry Staining and Cell Counting in Substantia Nigra Pars Compacta (SNpc)

After the EPM behavioural test, control and 6-OHDA lesioned rats with or without simvastatin administration were used for tyrosine hydroxylase (TH) staining. TH staining was performed as described in Yuan's study [31]. Briefly, endogenous peroxidase was quenched with 0.3% H_2_O_2_ (30 min). Non-specific binding was blocked with 1.5% normal goat serum (Vectastain rabbit IgG ABC kit) (60 min). This was followed by application of TH primary antibody (rabbit polyclonal anti-tyrosine hydroxylase, Millipore Corporation, AB152) at 1∶500 in blocking solution. The sections were incubated with the biotinylated anti-rabbit secondary antibody at 1∶200 (Vectastain rabbit IgG ABC kit) for 60 min. The horseradish peroxidase conjugate ABC (Vectastain rabbit IgG ABC kit) was applied for 60 min, followed by the nickel stock (DAB, Vector SK-4100). Intact dopaminergic cells that were round with clear nuclei or cytoplasm were counted; this analysis was carried out on five sections per animal through the SNpc anterior-posterior axis. The number of TH-positive cells was counted in 30 randomly selected fields. Data are means ± SE of values from three independent experiments.

### [^3^H] MK-801 Binding Autoradiography

After the EPM behavioural test, rats were sacrificed with an overdose of CO_2_ (carbon dioxide) between 0700 and 0900 hours in order to minimize the impact of circadian variation on binding density and the brains were immediately removed and frozen in liquid nitrogen. Coronal brain sections (14 um) were cut at −17°C with a cryotome (Clinicut cryostat; Bright Instruments) and thaw-mounted onto poly-L-lysine-coated microscope slides (Polysine™, Menzel GmbH & Co KG). Consecutive sections were used for the detection of the NMDA receptor binding site. Identification of neuroanatomical structures was performed according to a standard rat brain atlas [32]. [^3^H] MK-801 autoradiography was performed as described in our previous works [22]. Briefly, sections were preincubated for 2.5 h at room temperature in 30 mM N-2-hydroxyethyl piperazine-NO-2-ethanesulphonic acid (HEPES) buffer (pH 7.5), containing 100 mM glycine, 100 mM glutamate, 1 mM ethylenediaminetetraacetic acid (EDTA) and 20 nM [^3^H]MK-801. Non-specific binding was determined by incubating adjacent sections with [^3^H] MK-801 in the presence of 20 mM MK-801. Following incubation, sections were washed three times for 20 min each at 1°C in 30 mM HEPES containing 1 mM EDTA (pH 7.5).

### Quantification of [^3^H] MK-801 Binding

Quantification of binding sites was performed on a high-resolution Beta Imager (BioSpace, Paris, France) according to our previous study [22]. Briefly, sections were placed inside the detection chamber of the Beta Imager and scanned for 3.5 h at a high-resolution setting. The levels of bound radioactivity in the brain sections were directly determined by counting the number of β-particles emerging from the tissue sections, which was followed by analysis of the activity in the regions of interest using the Beta Vision Plus program (BioSpace). The radioligand binding signal was expressed in counts per minute per square millimetre (cpm/mm^2^), and a series of sections with known amounts of ligands were used as standards in all scans, which allowed the measurement of radioligand binding signals to be converted to nCi (nanocurie)/mg tissue equivalents. The [^3^H] MK-801 binding density in various brain regions was quantified by measuring the average density of each region in three to five adjacent brain sections.

### Cell Culture and Treatments

PC12 cell culture was performed as described in Rodriguez-Blanco's study [33]. Briefly, PC12 cells were routinely maintained in DMEM(Dulbecco's Modified Eagle Medium)supplemented with 5% fetal bovine serum, 10% horse serum, benzyl penicillin 100 U/ml, and streptomycin 100 mg/ml (Gibco). For all experiments, cells were seeded on the 96-well plates or 6-well plates at a density of 1.0×10^5^ cells/ml for 24 h. Three groups were treated with DMEM, 6-OHDA (100 uM), and 6-OHDA (100 uM)+simvastatin (0.6 ug/ml), respectively. For the determination of cell viability, 3-(4,5-dimethyl-2-thiazo-lyl)-2,5-diphenyl-2H-tetrazolium bromide (MTT) assay, glutamate concentration, and lactate dehydrogenase (LDH) release assay were conducted.

### MTT assay and Apoptotic Cells

The MTT assay was carried out with modifications according to Rodriguez's study [33] to measure the PC12 viability after 6-OHDA or 6-OHDA+simvastatin treatment. The results were expressed as a percentage of the control group. To measure apoptosis in this study, cells were stained with Hoechst 33342. Briefly, PC12 cells were seeded at a density of 1×10^5^ cells/well into 24-well plates. After incubation with 6-OHDA (100 uM) or 6-OHDA (100 uM)+simvastatin (0.6 ug/ml) for 24 h, cells were treated with Hoechst 33342 (10 mg/ml) (Sigma) for 20 min at 37°C in the dark. The cells were examined using an Olympus IX70 inverted fluorescence microscope. Ten randomly selected fields were acquired from each treatment and at least 500 cells were counted. PC12 apoptosis was also evaluated by flow cytometry using Annexin V-FITC (fluorescein isothiocyanate) (Bender MedSystems, Burlingame, CA): apoptotic cells display phosphatidylserine on the outside of the plasma membrane. Changes in phosphatidylserine asymmetry were analyzed by measuring Annexin V binding to the cell membrane.

### LDH Assay and Glutamate Measurement

Cell viability was also measured by determining the activity of LDH released into the medium [33]. After the 6-OHDA or 6-OHDA+simvastatin treatments, released LDH was measured, and cells were lysed to obtain total LDH. Measurement of total and released LDH activity was undertaken following specifications of the In vitro Toxicology Assay Kit LDH-based Tox-7 (Sigma-Aldrich, USA), and released LDH was normalized to total LDH. Data were represented as a percentage of LDH in the 6-OHDA group, which was designated as 100%. The concentration of glutamate was measured according to the Glutamate Assay Protocol (BioVision, USA).

### Protein Extraction, Subcellular Fractionation, and Western Blotting Analysis

After 6-OHDA or 6-OHDA+simvastatin treatment, cells were harvested by using cell scrapers and washing in ice-cold PBS, and lysed with two different ice-cold lysis buffers [33]. The supernatants were collected for protein determination by BCA (bicinchoninic acid) assay (Pierce, Inc., Rockford, IL, USA), and protein was run in NuPage Bis-Tris 10% gels (Invitrogen) and transferred to PVDF(polyvinylidene fluoride)membranes (Amersham Bioscience, Ltd., Buckinghamshire, UK). The membranes were blocked in 5% skim milk, 0.05% Tween 20, and Tris-buffered saline (TBS) for 1 h. PVDF membranes were incubated in primary antibodies: rabbit anti-TNF-a (1∶400), rabbit anti-matrix metalloproteinase-9 (MMP9) (1∶500), rabbit anti-NMDAR1(1∶800), or rabbit anti-β-actin (1∶1000) (all from Abcam, Cambridge, MA, USA), for overnight at 4°C. The next day, horseradish peroxidase-conjugated secondary antibodies (Calbiochem, San Diego, CA, USA) were applied. Peroxidase-conjugated streptavidin and substrate were used for detection. Negative controls were performed by omitting the primary antibodies. The images were analyzed using the NIH Image J software.

### Immunocytochemistry

Immunocytochemistry was performed and modified according to Iida's study [34]. After the nonspecific reaction was blocked with PBS containing 10% (wt/vol) bovine serum albumin (BSA), cells were incubated with the primary antibodies (anti-NMDAR1, 1∶ 200; anti-TNF-a, 1∶100; Abcam, Cambridge, MA, USA) in PBS containing 3% (wt/vol) BSA overnight. The next day, the secondary antibody (1∶200,Invitrogen, Carlsbad, CA, USA) was applied for 1 h. After the samples were washed three times with PBS, they were embedded in 200 ul Hoechst 33342 (concentration 10 ug/ml) for 5 minutes. The images were obtained using a Leica DMI 4000B microscope (Leica Corp.). Image analysis software Pro Plus 6.0 (Media Cybernetics Inc, Bethesda, USA) was applied to measure the intensity of NR1 and TNF-a receptors.

### Statistical Analysis

Data were expressed as mean ± SEM. Data related to [^3^H]MK-801 binding densities for each brain region, TH immunohistochemistry staining in the SNpc, MTT, LDH, Hoechst 33342, flow cytometry analysis, and protein quantification with western blot were analyzed using a one-way ANOVA (analysis of variance) followed by Tukey's post hoc analysis (Statistical Product and Service Solutions 15.0 program, Chicago, IL). Student's *t*-*test* was employed to determine the statistical significance of EPM test and immunocytochemistry staining. *p* values of less than 0.05 were regarded as statistically significant.

## Results

### Effects of 6-OHDA Lesion and Simvastatin on TH Immunohistochemistry Staining in the SNpc

**Figure 1:**
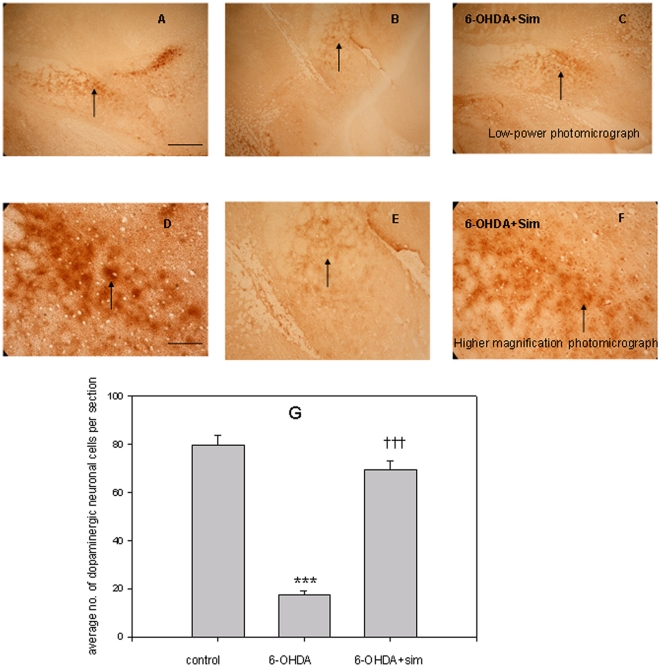
Effects of 6-OHDA lesion and simvastatin on TH immunohistochemistry staining in the SNpc. Figs. A, B, C shows TH staining in low-power photomicrograph in the SNpc of unlesioned, 6-OHDA-lesioned, and 6-OHDA-lesioned with simvastatin treatment groups, respectively. Bar = 450 µm. Figs. D, E, F shows TH staining at higher magnification photomicrograph in the SNpc of unlesioned, 6-OHDA-lesioned, and 6-OHDA-lesioned with simvastatin treated groups, respectively. Bar = 120 µm. Fig. 1G represents the average number of TH-positive dopaminergic neurons in the SNpc of unlesioned (control), 6-OHDA lesioned, and 6-OHDA lesioned with simvastatin treatment groups. The values represent mean ±SEM, n = 6–8. ***p<0.001, 6-OHDA group versus control group; ††† p<0.001, 6-OHDA+simvastatin group versus 6-OHDA group.

In Fig. 1 low-power photomicrograph Fig. 1A (scale bar, 450 µm) shows a coronal section of the unlesioned side through the midbrain, and Fig. 1B shows a lesioned section through the midbrain. Photomicrographs from Fig. 1D and Fig. 1E are taken from Fig. 1A and Fig. 1B at higher magnification, showing the unlesioned (Fig. 1D, left) and lesioned (Fig. 1E, right) side SNpc, respectively. After 6-OHDA MFB(medial forebrain bundle)lesion, cells in the SNpc displayed shrinkage. The typical TH immunoreactivity (Fig. 1D, scale bar, 120 µm) within the SNpc is located relative to the intact side; severe cell loss within the SNpc is ispilateral to the 6-OHDA MFB lesion (Fig. 1E). The injection of 6-OHDA produced a significant 78% decrease in the number of TH immuno-reactive dopaminergic neurons on the lesioned side of SNpc as compared to the control (F[_2]_, [18] = 142.77, *p*<0.001; Fig. 1G), whereas simvastatin treatment prevented this neuronal loss (Fig. 1C and Fig. 1F), keeping the number of TH immunoreactive neurons near control values (F[_2]_, [18] = 142.77, *p*<0.001; Fig. 1G).

### Effects of 6-OHDA Lesion and Simvastatin Treatment on [^3^H]MK-801 Binding

**Figure 2:**
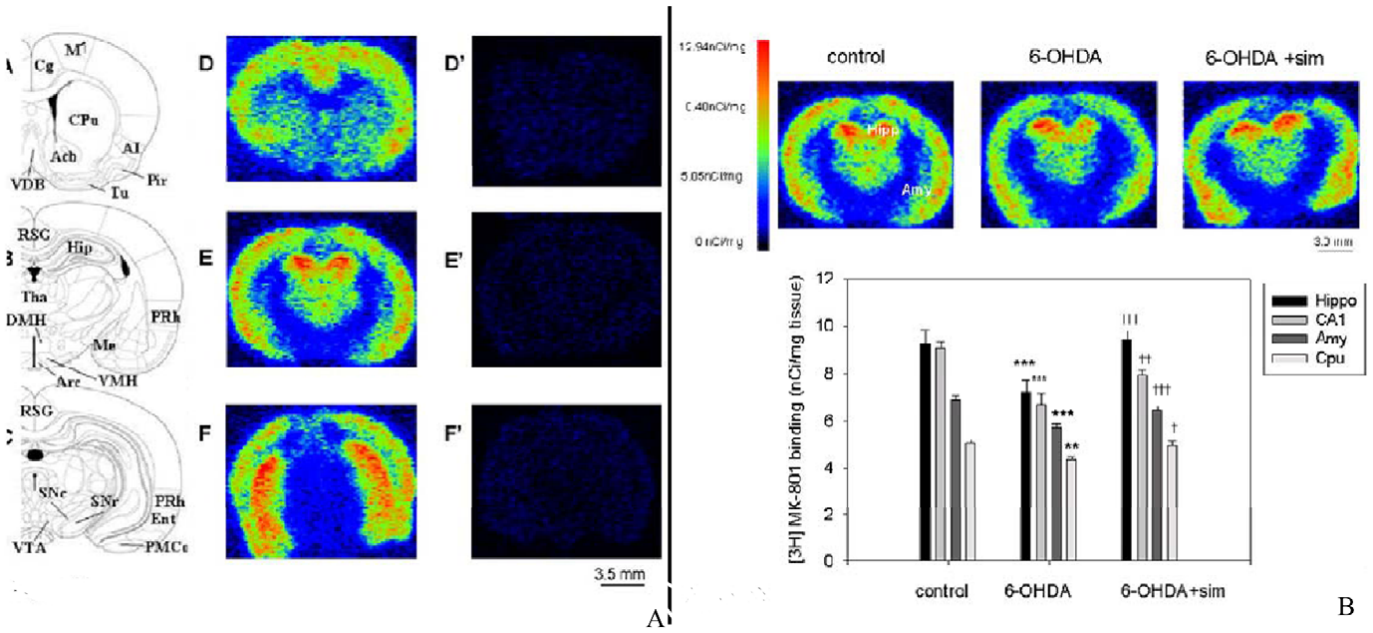
**2A.**[^3^H] MK-801 autoradiography depicts the expression of NMDA receptors in the rat brain. The maps of A, B and C are adopted from a rat brain atlas indicating the levels where the [^3^H]MK-801 binding density was measured. Autoradiographs (D, E, F) and (D', E', F') depict the expression of [^3^H]MK-801 binding and non-specific [^3^H]MK-801 binding at different rostro-caudal coronal levels of the rat brain. **2B.** Typical autoradiographs depict the expression of NMDA receptors in the hippocampus (Hipp) and amygdala (Amy) among control, 6-OHDA-lesioned rats, and 6-OHDA lesioned rats that also received simvastatin treatment. The bar chart shows the effects of chronic simvastatin treatment on [^3^H]MK-801 binding in the different groups of rat brain regions. *Note*: Units of measurement are in nCi/mg tissue. Data are means ± SEM. Asterisks indicate significant differences from control group (saline) and cross indicates significant differences between 6-OHDA rats and 6-OHDA with simvastatin-treated rats (n = 6–8, **p<0.01; ***p<0.001; †p<0.05; ††p<0.01; †††p<0.001, one-way ANOVA followed by Tukey's test).

Specific [^3^H]MK-801 binding was observed in most brain regions examined, and nonspecific binding was observed to be less than 5% (Fig. 2A). One way ANOVA revealed significant changes in [^3^H]MK-801 binding in the hippocampus (F[_2]_, [18] = 8.665), CA1 (F[_2]_, [18] = 7.486), amygdala (F[_2]_, [18] = 17.316) and caudate putamen (F[_2]_, [18] = 5.001) among 6-OHDA-lesioned rats. Specifically, Tukey's post-hoc analysis showed that three weeks after 6-OHDA lesion [^3^H]MK-801 binding was significantly decreased in the hippocampus (23%, p<0.001), CA1 region (26%, p<0.001), amygdala (18%, p<0.001) and caudate putamen (15%, p = 0.001) as compared to the controls (Fig. 2B). However, after three-week administration with simvastatin, [^3^H]MK-801 binding sites in these examined regions had clearly been restored to baseline levels. Specifically, simvastatin significantly increased [^3^H]MK-801 binding density in the hippocampus (31%, p<0.001), CA1 region (17%, p = 0.007), amygdala (18%, p<0.001) and caudate putamen (13%, p = 0.01) in comparison to the 6-OHDA lesioned PD rats (Fig. 2B). In addition, we did not detect [^3^H]MK-801 binding in the substantia nigra among either groups because the density was very low (not detectable), which is consistent with Araki's study [35].

### Anxiety Activity and its correlation with [^3^H]MK-801 binding

**Figure 3:**
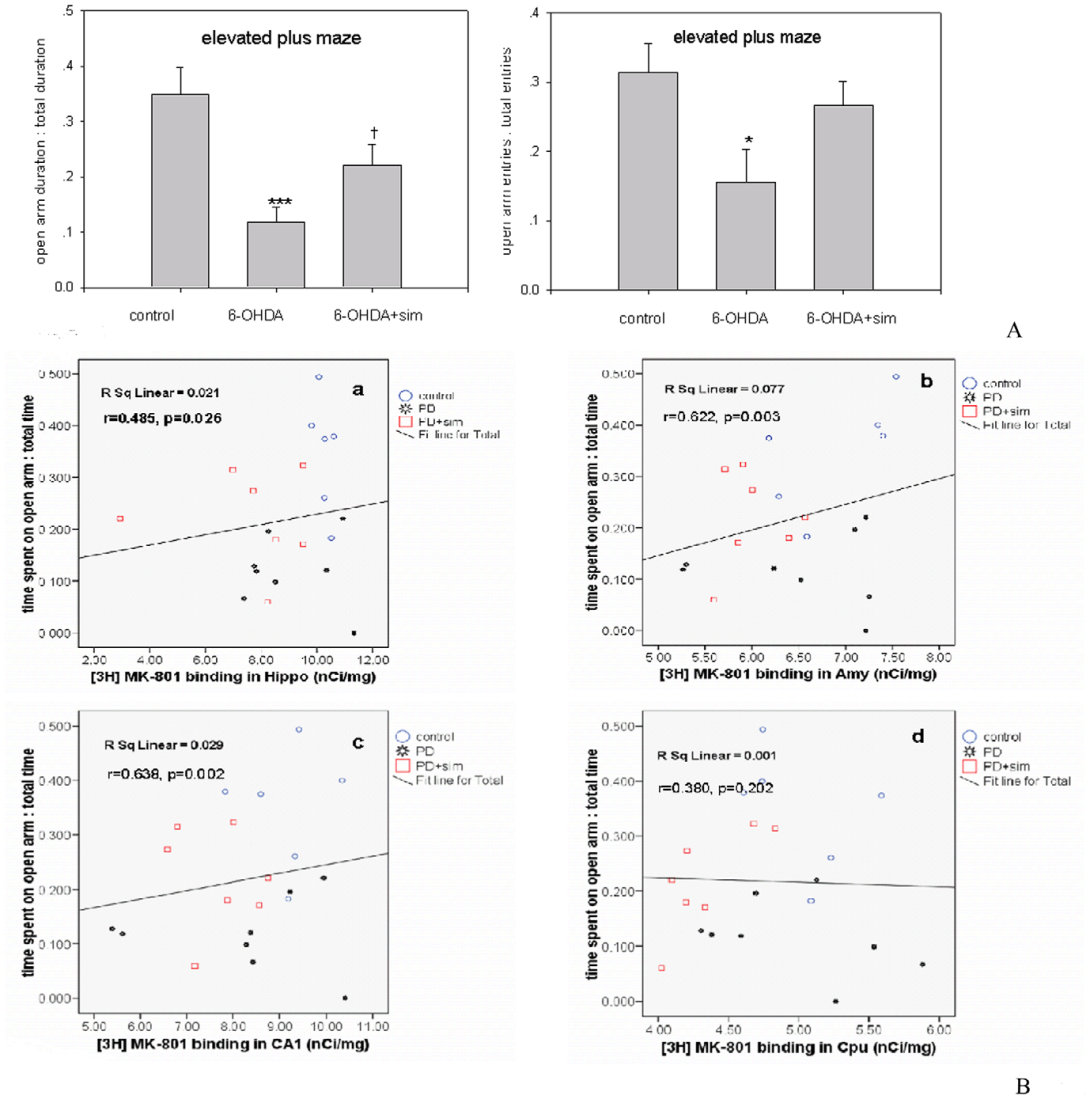
**3A.**Simvastatin ameliorates the anxiety of 6-OHDA rats in the EPM test. The graph shows the ratio of time spent in the open arms to total time and the ratio of open arm entries to total entries in the EPM. The parameters are expressed as a percentage of time spent in the open arms to the total time and open arm entries to total entries in the EPM. The values represent mean ± SEM, n = 6–8. †p<0.05, 6-OHDA group versus 6-OHDA+simvastatin group for open arm duration; ***p<0.001, 6-OHDA group versus control group for open arm duration; *p<0.05, 6-OHDA group versus control group for open entires. **3B.** Correlations between duration in the open arm of EPM and [^3^H]MK-801 binding density in brain regions. A significant positive correlation was identified between the [^3^H]MK-801 binding density in the hippocampus (r = 0.485 Pearson's correlation, p = 0.026), amygdala (r = 0.622, p = 0.003), CA1 (r = 0.638, p = 0.002), respectively, and the time spent in the open arm of the EPM.

Fig. 3A presents the anxiety-like behavior effect in the EPM test for control, 6-OHDA-lesion and 6-OHDA-lesion with simvastatin treatment groups. Student's *t*-test showed an obvious decrease (66%, Student *t*-test: *t* = 4.803, *p*<0.001) in the duration of open-arm activity in comparison to controls (Fig. 3A). When compared to 6-OHDA-lesion PD rats, simvastatin significantly restored the reduction in the duration of open-arm activity (86%, Student *t*-test: *t* = −2.422, *p* = 0.031). Student's *t*-test also showed an obvious decrease (49%, Student *t*-test: *t* = 2.688, *p* = 0.02, Fig. 3A) in the entries into the open arms in comparison to controls. When compared to 6-OHDA-lesion PD rats, simvastatin showed an increased tendency but not significant effect in the entries into the open arms (Student *t*-test: *t* = 2.072, *p* = 0.060, Fig. 3A). A significant positive correlation was identified between the [^3^H]MK-801 binding density in the hippocampus and the duration of time spent in the open arm (r = 0.485 Pearson's correlation, p = 0.026) in the EPM test (Fig. 3B). There were also significant correlations between the [^3^H] MK-801 binding density in the amygdala (r = 0.622, p = 0.003) and CA1 (r = 0.638, p = 0.002), respectively, with the duration of open-arm activity (Fig. 3B). However, no significant correlation was observed between [^3^H]MK-801 binding density in the caudate putamen and the duration of time spent in the open arm of EPM (r = 0.380, p = 0.202) (Fig. 3B).

### Effects of 6-OHDA and Simvastatin on PC12 Cell Viability and Apoptosis

**Figure 4:**
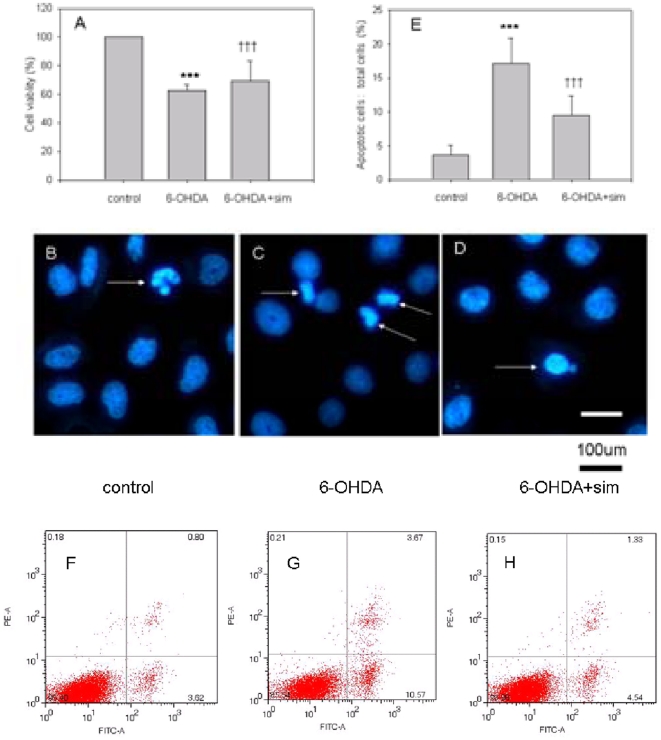
Simvastatin protected PC12 cells against 6-OHDA neurotoxicity. The MTT value in the 6-OHDA treated group was significantly reduced as compared with controls (***p<0.001, 6-OHDA *vs* controls, n = 9; Fig. 4A), but simvastatin upregulated this reduction (†††p<0.001, 6-OHDA *vs* 6-OHDA+sim, n = 9; Fig. 4A). Intact nuclei (blue Hoechst 33342 staining) and condensed/fragmented nuclei (bright blue Hoechst 33342 staining) were considered to be live and apoptotic cells, respectively (Fig. 4B, C, D). The exposure of the PC12 cultures to 6-OHDA (100 uM, 24 h) significantly increased the number of apoptotic cells by 4.75 times compared with controls (***p<0.001, 6-OHDA *vs* controls; Fig. 4E); however, simvastatin incubation significantly reduced this increase in the number of apoptotic cells (†††p<0.001, 6-OHDA *vs* 6-OHDA+sim; Fig. 4E; Bar = 100 µm). Apoptotic cells were further verified by flow cytometry analysis. The result showed that 6-OHDA induced profound apoptosis (4.59±0.9% *vs* 14.97±1.25%, controls *vs* 6-OHDA, p<0.01, n = 5; Fig. 4F and 6G) but simvastatin incubation attenuated this apoptotic death (14.97±1.25% *vs* 6.09±0.64%, 6-OHDA *vs* 6-OHDA+sim, p<0.01, n = 5; Fig. 4G and 4H). All the results are expressed as mean ± standard error of the mean.

The MTT value in the 6-OHDA treated group was significantly reduced compared with controls (F[_2]_, [26] = 580.791, ***p<0.001, 6-OHDA *vs* controls, n = 9; Fig. 4A), but simvastatin upregulated this reduction (F[_2]_, [26] = 580.791, †††p<0.001, 6-OHDA *vs* 6-OHDA+sim, n = 9; Fig. 4A). We examined the cultures exposed to 6-OHDA for the presence of apoptotic nuclei in PC12 cells using Hoechst 33342. Intact nuclei (blue Hoechst 33342 staining blue) and condensed/fragmented nuclei (bright blue Hoechst 33342 staining) were considered alive and apoptotic cells (Fig. 4B, C, D), respectively. The exposure of the PC12 cultures to 6-OHDA (100 uM, 24 h) significantly increased the number of apoptotic cells by 4.75 times compared with controls (F[_2]_, [26] = 316.785, ***p<0.001, 6-OHDA *vs* controls, n = 9; Fig. 4E); however, simvastatin incubation profoundly reduced this elevation in the number of apoptotic cells (F[_2]_, [26] = 316.785, †††p<0.001, 6-OHDA *vs* 6-OHDA+sim, n = 9; Fig. 4E). Apoptotic cells were further verified by flow cytometry analysis after being labeled with Annexin V. The result showed that 6-OHDA induced profound apoptosis (F[_2]_, [14] = 166.335, 4.59±0.9% *vs* 14.97±1.25%, controls *vs* 6-OHDA, p<0.01, n = 5; Fig. 4F,G) but simvastatin incubation attenuated this apoptotic death (F[_2]_, [14] = 166.335, 14.97±1.25% *vs* 6.09±0.64%, 6-OHDA *vs* 6-OHDA+sim, p<0.01, n = 5; Fig. 4G, H).

### Effects of 6-OHDA and Simvastatin on LDH and Glutamate

**Figure 5:**
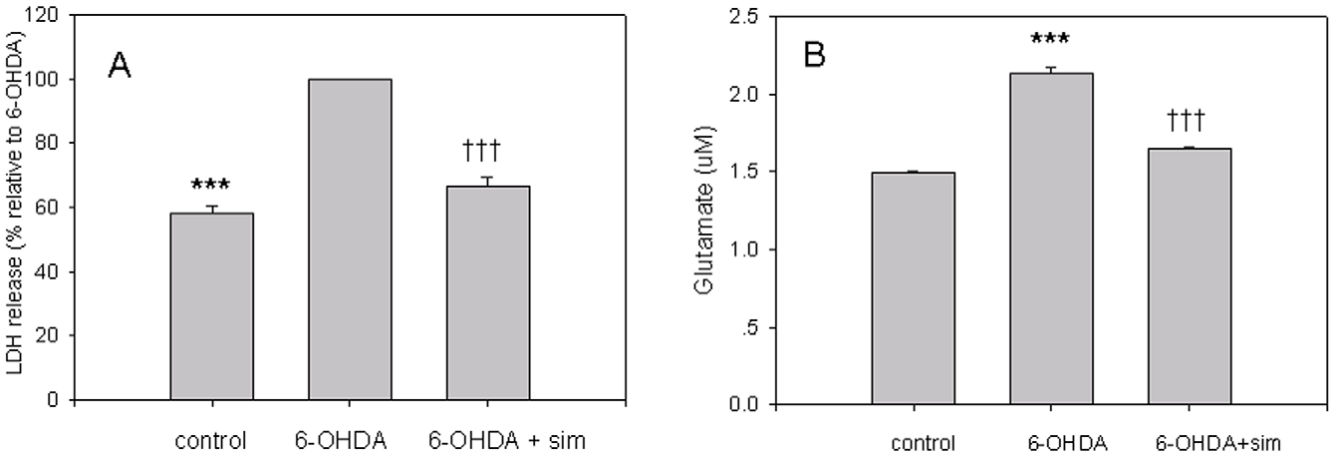
Simvastatin reduced 6-OHDA-induced LDH and glutamate. LDH in 6-OHDA incubated PC12 increased by 1.74 times compared with controls (***p<0.001, 6-OHDA *vs* controls, n = 9; Fig. 5A), but simvastatin incubation abolished this elevation (†††p<0.001, 6-OHDA *vs* 6-OHDA+sim, n = 9; Fig. 5A). In 6-OHDA incubated PC12, glutamate was increased by 1.43 times compared with controls (2.138±0.03 µm *vs* 1.49±0.01 µm, 6-OHDA *vs* controls, ***p<0.001, n = 9; Fig. 5B), but simvastatin treatment abolished this elevation (2.138±0.03 µm *vs* 1.64±0.01 µm, 6-OHDA *vs* 6-OHDA+sim, †††p<0.001, n = 9; Fig. 5B). All of the results are expressed as mean ± standard error of the mean.

LDH is released from the cells following membrane collapse, and the released LDH is usually considered a sign of late cell death [33]. Our result showed that LDH in 6-OHDA incubated PC12 increased by 1.74 times compared with controls (F[_2]_, [26] = 158.486, ***p<0.001, 6-OHDA *vs* controls, n = 9; Fig. 5A), but simvastatin incubation abolished this elevation (F[_2]_, [26] = 158.486, †††p<0.001, 6-OHDA *vs* 6-OHDA+sim, n = 9; Fig. 5A).Glutamate is the most abundant excitatory neurotransmitter and is recognized as an important sign of cell death. In 6-OHDA incubated PC12, glutamate increased by 1.43 times compared with controls (2.138±0.03 µm *vs* 1.49±0.01 µm, 6-OHDA *vs* controls, F[_2]_, [26] = 34.244, ***p<0.001, n = 9; Fig. 5B), but simvastatin incubation abolished this elevation (2.138±0.03 µm *vs* 1.64±0.01 µm, 6-OHDA *vs* 6-OHDA+sim, F[_2]_, [26] = 34.244, †††p<0.001, n = 9; Fig. 5B), demonstrating a significant neuroprotection against PD in this in vitro model.

### Simvastatin Regulates the Levels of NMDANR1 Receptors, TNF-a MMP9 in 6-OHDA-treated PC12 cells using Western blot analysis

**Figure 6:**
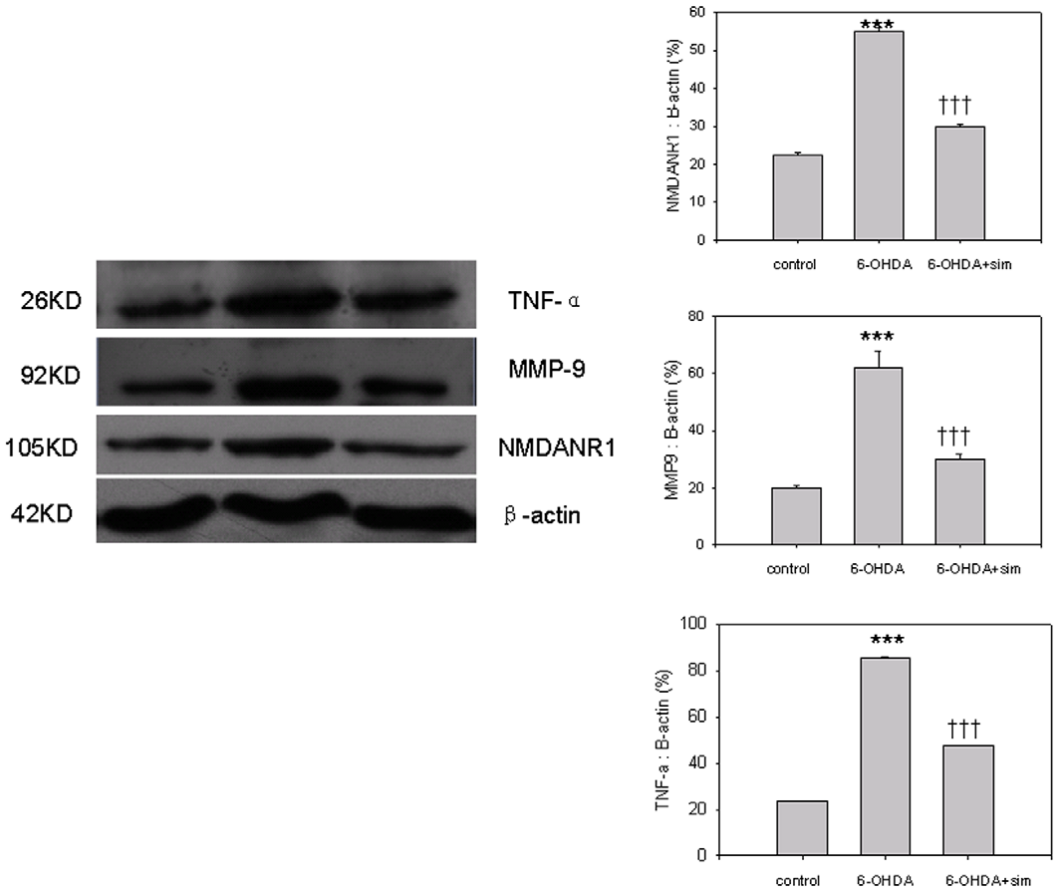
Simvastatin reduced 6-OHDA medicated elevations of NMDANR1 receptors, TNF-a, and MMP9. 6-OHDA incubation pronouncedly increased the NR1 receptors compared with controls (***p<0.001, 6-OHDA *vs* controls, n = 6–9); while this elevation was significantly abolished following simvastatin treatment (†††p<0.001, 6-OHDA *vs* 6-OHDA+sim, n = 6–9). Compared with controls, 6-OHDA produced significant increases in the total amount of TNF-a and MMP9 (***p<0.001, 6-OHDA *vs* controls, n = 6–9); while these increases were prevented by simvastatin treatment (†††p<0.001, 6-OHDA *vs* 6-OHDA + sim, n = 6–9). All the results are expressed as mean ± standard error of the mean.

6-OHDA incubation pronouncedly increased levels of NR1 receptors as compared with controls (F[_2]_, [21] = 142.568, ***p<0.001, 6-OHDA *vs* controls, n = 6–9, Fig. 6), but this elevation was significantly abolished following simvastatin treatment (F[_2]_, [21] = 142.568, †††p<0.001, 6-OHDA *vs* 6-OHDA+sim, n = 6–9, Fig. 6). To explore whether the modulation of NR1 receptors following simvastatin treatment is correlated with anti-inflammatory responses, the levels of inflammatory mediators TNF-a and MMP9 were also determined by western blot. Compared with controls, 6-OHDA produced significant increases in the total amount of TNF- a and MMP9 (F[_2]_, [21] = 284.56, ***p<0.001, 6-OHDA *vs* controls, n = 6–9, Fig. 6); while these increases were prevented by simvastatin treatment (F[_2]_, [21] = 284.56, †††p<0.001, 6-OHDA *vs* 6-OHDA+sim, n = 6–9, Fig. 6).

### Simvastatin attenuates the protein and size of NMDANR1 and TNF-a in 6-OHDA-treated PC12 cells

**Figure 7:**
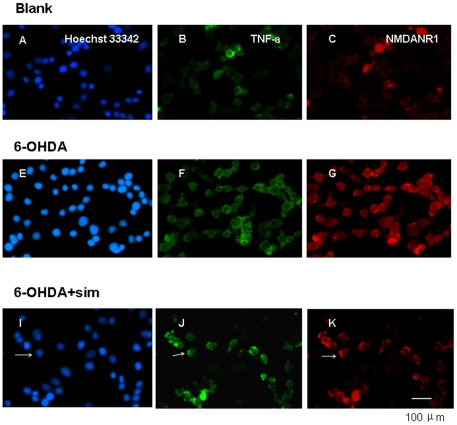
6-OHDA increased synaptic cluster density and number of clusters NR1 receptors and TNF-a, and the upregulation was abolished after simvastatin treatment. Arrows in ***I, J, K*** indicate nuclear, TNF-a, and NR1, respectively. PC12 cultures double-labeled for NR1 (red, ***C,G,K***) and TNF-a (green, ***B,F,J***); Hoechst 33342 indicates nuclear staining (blue, ***A, E, I***). 6-OHDA treatment significantly increased the density of NR1 (***G***) and TNF-a clusters (***F***), and the elevated density was abolished by simvastatin treatment (***K, J*** for NR1 and TNF-a, respectively). A significant difference in the density of NR1 and TNF-a was observed among control, 6-OHDA, and 6-OHDA+sim groups (*p*<0.05, control *vs* 6-OHDA; *p*<0.05, 6-OHDA *vs 6-OHDA*+sim; *n* = 9–12; Student's *t* test). All the results are expressed as means ± standard error of the mean. Scale bars: 100 µm.

To further examine whether a simvastatin-induced decrease of NMDANR1 receptors in the postsynaptic membrane may be associated with levels of inflammatory cytokine TNF-a, 6-OHDA-treated PC12 cells treated with simvastatin was subjected to immunocytochemical staining. Numerous punctate clusters containing NR1 immunoreactivity were found among synaptic cluster (Fig. 7C). We compared the density and location of NMDANR1 receptor clusters in sets of randomly selected control, 6-OHDA-treated, and 6-OHDA+simvastatin treated PC12 cells. As shown in Fig. 7G, the quantification confirmed that the exposure of PC12 to 6-OHDA for 24 hrs greatly increased the density of NR1 clustering at the synaptic cleft (*p*<0.05, *n* = 9–12, control *vs* 6-OHDA), which is consistent with the results of western blot analysis. However, incubation with simvastation significantly abolished this up-regulation of NR1 clustering in the synaptic arbors (*p*<0.05, 6-OHDA *vs 6-OHDA*+sim, *n* = 9–12; Fig. 7K). The simvastatin-mediated decrease of NR1 clusters to synaptic sites suggests that NMDAR transport along the dendrite may be altered or, alternatively, receptor protein stabilization may occur. In addition, the quantification of TNF-a revealed a similar result: TNF-a was present in the dendrites of PC12 cells and increased after 24-hr 6-OHDA exposure (*p*<0.05, *n* = 9–12, control *vs* 6-OHDA; Fig. 7B and Fig. 7F). This elevation of TNF-a was decreased following simvastatin treatment (*p*<0.05, 6-OHDA *vs 6-OHDA*+sim, *n* = 9–12; Fig. 7J). The similarities in the observed similar patterns of NMDANR1 receptors and TNF-a expression in the PC12 cultures suggest that the changes of NR1 receptors and TNF-a are associated with simvastatin treatment.

## Discussion

In this study, the pronounced reduction of TH immunoreactivity and decreased numbers of TH-immunoreactive dopaminergic neurons in the SNpc of the 6-OHDA-lesioned side were observed, demonstrating an obvious dopaminergic neuronal degeneration and complete nerve terminal denervation, which are necessary for a successful PD animal model. Our study also shows that simvastatin prevented 6-OHDA induced dopaminergic neuronal loss, strongly implying that simvastatin would provide a neuroprotective effect in PD. This result is consistent with Ghosh and Hernandez-Romero's study, which demonstrated that statins slowed down dopaminergic degeneration and may be of therapeutic benefit for PD patients [27], [28]. It was shown that, in the EPM, 6-OHDA lesioned rats spent less time in the open arm and an obvious decrease in the entries into the open arm compared to the controls (Fig. 3A), reflecting 6-OHDA lesion-mediated anxiety-like behaviour. Our result is consistent with Tadaiesky and Espejo's studies demonstrating that 6-OHDA lesioned PD rats showed increased anxiety-like activityes [36], [37]. Increasing evidence indicates that before the motor features occur, Parkinson's patients usually present one or more nonmotor symptoms, typically as cognitive and neuropsychiatric dysfunctions [38]. Among those neuropsychiatric dysfunctions, anxiety is very common in PD patients, with prevalence rates of up to 30% depending on the criteria used [39]. Therefore, it is imperative to explore the mechanisms underlying the anxiety-like activity. The current animal study directly reflects this neuropsychiatric profile in clinical PD patients and suggests possible mechanisms.

Our study showed that 6-OHDA lesion in the MFB reduced NMDA receptor expression in the brain regions examined (Fig. 2B), which is similar to other studies, demonstrating that NMDA receptors or its subunits were decreased in the brain following unilateral dopamine depletion [40], [41]. However, how and why NMDA receptors were decreased following the 6-OHDA MFB lesion remains to be conclusively determined. Several lines of evidence demonstrated that striatal dopaminergic denervation resulted in increased afferent glutamatergic input [42], [43]; therefore, we hypothesize that the downregulation of NMDA receptors in 6-OHDA lesioned rat brain is due to increased levels of striatal glutamate following nigrostriatal dopamine denervation. Notably, we cannot preclude that the downregulation of NMDA receptors in the examined regions may reflect NMDA hypo-innervations following 6-OHDA lesion. However, the precise reasons behind this phenomenon remain to be determined.

It is well documented that NMDA receptors in the brain have a close correlation with anxiety-like activity. In NMDA NR3B (N-methyl,D-aspartate receptor subunit 3B) receptor knockout mice, pronounced decrease in activity and increase in anxiety-like behaviour were observed, suggesting that the function of the NMDA receptor directly contributes to anxiety processing [44]. Similarly, Johnson and Shekhar found that anxiety-like responses in rats were regulated by the NMDA NR1 subunit and NMDA receptor antagonists [8]. Our current study showed that the NMDA receptor was significantly decreased in the striatum, hippocampus, CA1 and amygdala brain regions of the 6-OHDA lesioned side. This robust downregulation of NMDA receptor in the examined brain regions of 6-OHDA lesioned rats correlated with longer duration of open-arm activity in the EPM (Fig. 3B), strongly suggesting that the NMDA receptor hypofunction in these brain regions explains, at least partially, the anxiety-like activity in 6-OHDA induced PD rats. This hypothesis could also be supported by the facts that the altered levels of NMDA receptors in the hippocampus and amygdala directly influence anxiety behaviours [10], [22].

In the current study, as our previous work and Byrnes' study [22], [30], the elevated plus maze test was used to measure the anxiety of rats following 6-OHDA lesion and simvatatin treatment. Two indicators, the duration spent in the open arm and entries into the open arm, were applied to evaluate the anxiety of rats. Increased time, and/or entries traveled in the open arms of the EPM are interpreted as reduced anxiety-like behavior. Our data showed that when compared to 6-OHDA-lesion PD rats, simvastatin only produced an increased tendency but not significant effect in the entries into the open arms (p = 0.060, Fig. 3A). This result may be due to either the small numbers of rats used in this study, or the rats being reluctant to move following the 6-OHDA lesion. This increased tendency in the entries into the open arms following simvastatin treatment, at least partially, indicated that simvastatin could attenuate the 6-OHDA induced anxiety. Moreover, our results (Fig. 3A) also showed that simvastatin administration profoundly increased the reduced time spent by 6-OHDA lesioned rats in the open arm of the EPM (Fig. 3A), reflecting the ability of simvastatin to produced a pronounced anxiolytic-like effect. Consistent with our hypothesis, in a retrospective cohort investigation Starr found that statins obviously ameliorated anxiety disorder from in people aged 11–80 [45]. Increasing evidence shows that statins have been used clinically to restore the cognitive deficits in different neurodegenerative disorders such as PD, AD and vascular dementia [46], [47], and the cumulative reduction in the levels of anxiety risk for patients is independent of the statins' cholesterol-lowering effect [48]. However, how statins affect anxiety and the underlying mechanisms remain unclear. This study showed that the down-regulation of NMDA receptors in these examined regions was obviously restored following simvastatin administration. The present study is consistent with our previous observation in which simvastatin upregulated NMDA receptors in the naïve rat brain, and further validates our proposal that simvastain may exhibit NMDA antagonist-like effects [22]. Our results demonstrated that the upregulation of NMDA receptors in the hippocampus, CA1 and amygdala following simvastatin treatment had a significant positive correlation with the time spent in the open arm of the EPM (Fig. 3B), implying that simvastatin ameliorated anxiety behaviour in 6-OHDA lesioned rats via NMDA receptor modulation. Because previous studies have found that simvastatin affected dopamine levels as well as its metabolism in vivo [20], and because there exists a close interaction between the regulation of NMDA receptors and the dopaminergic system [49], [50], it is reasonable to speculate that simvastatin may exhibit an anxiolytic-like activity in 6-OHDA-lesioned rats by modulating the expression of NMDA receptors in the examined brain regions or influencing the interaction of NMDA receptors and the central dopaminergic system.

To explore the effects of simvastatin on PD in an in vitro model, 6-OHDA treated PC12 cells, an accepted PD in vitro model, were used in this study. The 6-OHDA incubated PC12 cultures exhibited an obvious decrease of cell viability and increased apoptosis (Fig. 4), indicating the establishment of a successful in vitro PD model. However, pre-incubation with simvastatin reduced cell viability and increased apoptosis, as determined using Hoechst 33342 and flow cytometry analysis. In addition, our results showed that LDH and glutamate were significantly increased in 6-OHDA-induced PC12 cells. These elevations were obviously prevented after simvastatin incubation, demonstrating that simvastatin induced pronounced neuroprotective effects. PC12 cells mainly express the functional NR1 receptor; therefore NR1 was chosen to detect the effects of 6-OHDA neurotoxicity and simvastatin in this study. It has been shown that the elevation of NMDA receptors is closely correlated with inflammatory responses and induced neuronal death [51], [52], [53]. In the current study, the increased NR1 expression and excitatory glutamate concentration were observed following 6-OHDA incubation (Figs. 5 and Fig. 6). This 6-OHDA induced elevation of glutamate excessively activated NMDANR1 expression, which further aggravated PC12 damage [54] and may have increased the susceptibilityof PC12 cells to excitotoxicity. However, the addition of simvastatin significantly abolished this elevation of NR1 and glutamate as well as the reduction in PC12 cell death. Considering that the elevation of NR1 and glutamate will lead to excitotoxicity and neuronal cell death, it is reasonable to speculate that in the current study simvastatin prevented PC12 cell death, at least partially, by protecting against NR1-induced excitotoxicity. This result is similar to Wang's study, showing that the upregulation of NR1 was correlated with neuronal cell death and abolishing this NR1 elevation prevented neuronal loss [55]. Interestingly, we observed that the changes of NMDA receptors following 6-OHDA and simvastatin treatment in vivo and in vitro PD models are contrary. These contrasting results may be that in vivo PD model the animals responded with auto-regulation to dopaminergic damage; while in vitro PD model only PC12 cells react to micro-environment changes following 6-OHDA and simvastatin treatment. However, the precise mechanisms need further study.

To explore whether inflammatory mediators in PC12 cells changed following 6-OHDA and simvastatin treatment, we measured the expression of TNF-a and MMP9. Our study showed increased expression of TNF-a and MMP9 in 6-OHDA-induced PC12 cells (Fig. 6), implying that these inflammatory mediators affected NMDA receptors expression. The elevation of NR1 and TNF-a and MMP9 was significantly abolished following simvastatin treatment, strongly suggesting a direct anti-inflammatory property of simvastatin through NMDA receptor modulation. The current result is consistent with several lines of evidence showing that the regulation of NMDA receptors is directly correlated with inflammatory mediators TNF-a and MMPs in pathological brain processes, including the mediation of neuronal death [56], [57], [58]. To further verify that the alteration of NMDA receptors is associated with inflammatory cytokine TNF-a, we focused specifically on 6-OHDA-treated PC12 expressing NR1 protein and analyzed the pattern and distribution of the punctate extranuclear immunostaining of TNF-a proteins presenting along dendrites. We detected a significant increase in NR1 protein clusters after 6-OHDA exposure; this increase was abolished following simvastatin treatment, whereas TNF-a proteins displayed a similar pattern after 6-OHDA neurotoxicity and simvastatin treatment (Fig. 7). The changed trend of TNF-a and NR1 proteins in our study (Fig. 7) indicated that NR1 proteins were closely associated with inflammatory cytokine TNF-a following 6-OHDA and simvastatin treatment. This result is consistent with other studies showing that pro-inflammatory mediator TNF-a is involved in simvastatin-mediated neuroprotection and associated with the altered expression of NMDA receptors [59]. To the best of our knowledge, this is the first attempt to describe the TNF-a and NR1 in PC12 and their similar changes in expression following inflammation.

In summary, our study presents the first evidence demonstrating the effects of simvastatin on NMDA receptors in the brain of 6-OHDA-lesioned rats and reveals an NMDA-modulatory effect, providing an exciting new paradigm to ameliorate anxiety-like activity in PD. Based on the current results, we reasonably speculate that the improvement in anxiety-like activity due to chronic treatment with simvastatin in 6-OHDA-lesioned rats is partially correlated with a reversal of the declined in NMDA receptors expression. Through in vitro and in vivo studies, our results strongly demonstrated that simvastatin provided robust neuroprotection against dopaminergic neurodegeneration, partially via NMDA receptor mediated anti-inflammatory mechanisms such as regulating TNF-a and MMP9. Although it is not a complete phenocopy of human disease, this 6-OHDA-mediated in vivo or in vitro PD models provides a useful means to study the pathomechanisms of clinical PD patients, as the models recapitulates many of the hallmarks of PD. A better understanding of the roles and relationships among statins, NMDA, and the dopaminergic system may open new perspectives for the statin family in the modulation of psycho-neurodegenerative disorders such as PD.
